# Supplementary material for: Effects of arthropod inquilines on growth and reproductive effort among metacommunities of the purple pitcher plant (Sarracenia purpurea var. montana)
Source: PLoS One. 2020 May 8;15(5):e0232835. doi: 10.1371/journal.pone.0232835 (PMC7209241; doi:10.1371/journal.pone.0232835)
Supplement: Attachment — Submitted filename: Review of Hale Revision.pdf [file pone.0232835.s002.pdf]

This manuscript is greatly improved in terms of the availability of their results. I worked on making up a table:

	a		b	
Hypothesis	2015	2016	2015	2016
1	r^2 =	ns	ns	r^2 = ?
2	ns	r^2 =	ns	ns
3	ns	?	r^2 =	?
4	ns	?	ns	?

If one of the 16 hypotheses were true, I then tried to enter the effect size into the table. I found filling in a significant effect or not (ns) possible but tedious for H1 and H2, but not clear for subsequent entries. What was difficult or not possible to decipher was the effect size of the predictor variables (inquiline abundance and diversity) they sought to test. My doctoral professor drilled into me "I grant you there is a significant effect, but is it substantial?" With a sample size of 380 and a 1-tailed test (their hypothesis is false if there is either no significant or a negative correlation), r^2 can be as low as 4.4% and still be "significant." The major contribution of this paper is in the results since the discussion is a concatenation of equivocation (caveat lector) – none of it wrong, because each conclusion is qualified but really summing to: The number or diversity of four arthropod inquiline species can have some, probably small, effect on host plant vegetative production or flowering in some years in some bogs at some times of the year. Were I writing a review paper on pitcher-plant ecology, this statement is how I would summarize this entire paper. Even this summary would depend on how I could fill in the table above. In short, the import of this paper remains in their data from observations in nature, replicated among bogs, among times of year, and between years, not in the discussion of their results.
