# Supplementary material for: Effects of arthropod inquilines on growth and reproductive effort among metacommunities of the purple pitcher plant (Sarracenia purpurea var. montana)
Source: PLoS One. 2020 May 8;15(5):e0232835. doi: 10.1371/journal.pone.0232835 (PMC7209241; doi:10.1371/journal.pone.0232835)
Supplement: Attachment — Submitted filename: Response to reviewers.docx [file pone.0232835.s001.docx]

*Comments of Dr. Etges:*

*The comments of the external referee are detailed and constructive. I too felt somewhat confused by the presentation of the statistical analyses and worried about the confidence in the regression results that seemed to be month-specific. With just two seasons of data, it is not surprising that such variation between months was found. I would have predicted a larger effect of phenology given that these organisms overwinter in the pitchers. After thawing, I would think there could be similar community trajectories over summer that are dependent on rainfall. I see no discussion of local precipitation in your analyses.*

We, too, expect communities to change over time, but such changes would be due to succession. We explicitly controlled for community age and, thereby, successional stage by sampling pitchers that had been opened less than one month. Our goal in sampling repeated months was to increase replication, though as it turned out we were not able to sample at all sites in all months, reducing by nearly half the number of sites with three months of sampling. This latter issue complicates our use of all three months’ data in the analyses of community traits, as we discuss below, and we now use rosette averages in our analyses.

Regarding rainfall, we report annual rainfall estimates in Table 1 and have now included estimates for both 2015 and 2016. 2016 was considerably drier than 2015 and many pitchers were empty when we sampled them. We now briefly make a connection between annual rainfall and flowering rates in the Results lines 344-347.

*The manuscript needs tightening with a clear and more direct connection between each of the statistical results and their implications for testing your original hypotheses. The data you have collected are valuable, and a better organized MS will help to attract readers to this study.*

In response to comments by both reviewers, we have made our hypotheses more explicit at the end of the Introduction (lines 118-137) and have numbered the hypotheses. Further, we have completely revised our analysis of the data. In general, the two reviews emphasized that we needed better link between the hypotheses and the tests, and that our analyses needed to be more clearly presented. Previously, we reported partial regression coefficients and F statistics that did not test marginal effects. We have modified our evaluation of fixed effects to use a model fitting approach – specifically, comparison of model AIC values to identify which terms are included in the model that explains the most variance in the data. While this method does not allow us to report the amount of variance explained by individual terms in the full model, it allows us to more clearly present which variables are important. These tabular results are coupled with figures showing the marginal effects that test our hypotheses.

*On a minor point, please correct the spelling of symbiont: "Symboint" appears in the title and several other places in the text.*

We have corrected the spelling of ‘symbiont’ in the first sentence of the Discussion and have removed the term from the title.

2. *We note that you have stated that you will provide repository information for your data at acceptance. Should your manuscript be accepted for publication, we will hold it until you provide the relevant accession numbers or DOIs necessary to access your data. If you wish to make changes to your Data Availability statement, please describe these changes in your cover letter and we will update your Data Availability statement to reflect the information you provide.*

Because of the cost of hosting data at Dryad, we are holding off on publishing the data there until the manuscript is accepted. However, the data are uploaded and are available for review at https://datadryad.org/stash/share/9Z3gogbGOlzisvv1uypqbG2ooz05TXM2keJw9scMcEc

REVIEWER #1

**Comments to the Author**

*3. The experiments were performed adequately given that they represent field experiments; the statistics are unclear at best and in at least two instances, there is inconsistency between the significance claimed and the ratio of the parameter estimate and its standard error; Tables 4 & 6 appear to report the results of multiple regression, an approach that does not correct for fluid volume (a continuous variable) or site (a discrete variable). Full statistical tables as SI would have helped here. Due to large sample sizes, some significant correlations are low so that consistently reporting coefficients of determination or % reduction in total sum of squares would help the reader decide whether there was a substantive as well as statistically significant result.*

All of our multiple regressions (Tables 4-5) include fluid volume and site as fixed effects, so we controlled statistically for those variables. As the reviewer suggests, we reported the F values associated with individual terms in the full model, as opposed to reporting F values associated with Type III Sums of Squares.

We have reanalyzed the data to use a model comparison approach, evaluating the AIC when a model with the independent variable of interest (e.g., flowered/not flowered in Table 4) is included versus when not included. This approach is, in our understanding, comparable to evaluating Type III SS. Comparison of AIC values, while not identical to the R^2^ or % change in SS, is comparable to such values because the magnitude of difference in AIC value between models correlates with the magnitude of the effect of the term that differs between them. Further, AIC comparison simplified presentation of the statistics in tables.

We also acknowledge that some of the terms in the best-fitting models have very small effects (e.g., Fig. 1), and we have revised our discussion to limit our inference from these results.

*5. English was good, but the manuscript was muddled due to an indirect approach to the main hypotheses. The line numbers petered out towards the end of the manuscript.*

We have re-inserted page numbers to assist with review and have ensured that they continue through the references. *6. The authors appeared to be very careful to (A) not reveal the coordinates of their localities due to the lamentable, but very real likelihood of poaching. They might give the total latitudinal and altitudinal range of their study to the nearest degree of longitude and 200m of elevation and (B) provide specific agency connections so other, legitimate researchers could seek permission to use their study sites.*

We have included the lat/long range as well as the elevational range to lines 140-141. We have added a list of landowners/permitting agencies to the acknowledgements so future researchers can contact appropriate agencies for access. *7. Data reporting: I expect the raw data to be archived, but there was no obvious mention of plans to do so in the MS. Full statistical tables presented as supplemental information would* *have alleviated or increased my concern about statistical handling of the data.*

The data are available on Dryad, but are not yet published. Upon official acceptance, we will publish the data. This information was provided upon initial submission, but may not have been visible to the reviewer. The Reviewer URL is https://datadryad.org/stash/share/9Z3gogbGOlzisvv1uypqbG2ooz05TXM2keJw9scMcEc

Comments
I. Motivation for this study

*As stated in the introduction, the motivation for this paper puts eight hypotheses (H’s in my analysis) at risk (lines 124-131):
Growth (= leaf production) increases with (H1) abundance and (H2) diversity of inquilines.
(H1) From top of Table 4, this hypothesis is supported in only one of three months in 2015 and not in any of the three months of 2016. For August, 2015, the reported P-value for Beta-1 is 0.005 but Beta-1 divided by its standard error is <1.96 and would appear not significant – some explanation in needed here.
(H2) From the bottom of Table 4, this hypothesis is supported by results from 2015 June and July, but not August and not by results from 2016. For July 2015, the reported P-value for Beta-1 is 0.021, but Beta-1 divided by its standard error is <<1.96 and would appear not significant - again, some explanation is needed here.
The middle of Table 4 has nothing to do with the stated hypotheses. If a significant effect of inquiline diversity were found, then testing for species richness or equitability would make sense for those comparisons where diversity did have an effect, but that is not the way the hypotheses are laid out or the results presented.

Reproductive effort (= number of flowers) increases with (H3) abundance and (H4) diversity of inquilines.
(H3) & (H4) From Table 6, top & bottom, respectively, neither hypothesis is supported in any month in either 2015 or 2016.
The middle of Table 6 has nothing to do with the stated hypotheses*

In response to the reviewer’s comments that the species richness data are not relevant, we would like to clarify that when we hypothesized that community diversity would vary with reproductive effort, growth, population size, and population isolation, we meant diversity broadly. We consider species richness and species evenness (Shannon’s H) both to be measures of diversity. From the comments, the reviewer appears to consider Shannon’s H the diversity measure and richness something different. Because richness and Shannon’s H are positively correlated, and because the results are already difficult to process, we agree with the reviewer that the richness data are unnecessary. We will leave references to the range of richness in the Species Composition section of the Results but remove the statistical analyses.

We have numbered our hypotheses in the last two paragraphs of the Introduction and reference those numbers in the appropriate data tables (Tables 3 and 4) and figures (3-4). We count four hypotheses (H1-H4) that each make two predictions (a and b):

H1: Inquiline communities affect vegetative growth.

H1a: Inquiline communities, measured as total inquiline abundance, will positively affect number of pitchers produced in a season.

H1b: Inquiline communities, measured a species evenness (Shannon’s H), will positively affect number of pitchers produced in a season.

H2: Inquiline communities affect flower production.

H2a: Inquiline communities, measured as total inquiline abundance, will be greater among rosettes that flower than among those that do not.

H2b: Inquiline communities, measured as species evenness, will be greater among rosettes that flower than among those that do not.

H3: As the number of plants in a population increases, so should abundance of inquilines and inquiline diversity.

H3a: Inquiline communities, measured as total inquiline abundance, will increase with the number of plants in a population.

H3b: Inquiline communities, measured a species evenness (Shannon’s H), will increase with the number of plants in a population.

H4: As the distance between populations (=isolation) increases, so abundance of inquilines and inquiline diversity should decrease.

H4a: Inquiline communities, measured as total inquiline abundance, will decrease as distance between populations increases.

H4b: Inquiline communities, measured a species evenness (Shannon’s H), will decrease as distance between populations increases.

The first two hypotheses are explained within our first objective and the second two hypotheses within our second objective (Lines 120-136). The eight hypotheses are listed in the text as H1a,b through H4a,b.

In light of our reanalysis of the data (see explanation of model-fitting approach, above), we no longer present the data by month. Instead, we have averaged across month. Our more subjective approach to evaluating main effects in the original manuscript was problematic and the new analyses solve these problems. *The size of the inquiline population (= number of inquilines in a leaf) (H5) increases with host population size and (H6) decreases with distance to nearest neighbor (= site). The pertinent results are provided without table or illustration in the first paragraph of “Population size and isolation” (The lines are not numbered in this section). Neither hypothesis is supported.*

*The diversity of the inquiline population (H7) increases with host population size and (H8) decreases with distance to nearest neighbor. The pertinent results are provided without table or illustration in the second paragraph of “Population size and isolation.” H7 is supported; H8 is not.*

In the original manuscript, we cited Figure 2 in the first sentence of this section (current line 364), and the populations in Figure 2 were sorted left to right from smallest to largest population. We have reorganized the results section and now present this figure as Fig 3 in the section Population Size and Isolation, to make the link clearer. We provided the rho statistics and the associated p values. We are grateful to the reviewer for pointing out that we misinterpreted our results. There was a positive correlation between population size and Shannon’s H, indicating that populations with more plants maintain more diverse communities. This correlation held when we evaluated Shannon’s H averaged across pitchers at a site, but not when we calculated a single H based on all organisms in all pitchers at a site. This is discussed in line 436-443 of the Discussion and the corrected Results are presented in lines 363-365 and 378-372. *What is needed here is a clear path to the tests of the staged hypotheses and then a discussion of what the results of those results mean, uncluttered with tangential material that reflects mainly ad hoc “what if’s.” The authors make a good case for pursuing the main hypotheses in the introduction and then muddle their results with other information that is incidental to the stated hypotheses to be tested, making for tedious reading and distracting from their primary mission.*

We thank Reviewer 1 for the careful reading of the paper and we have made major revisions to how the material is presented. The incidental information is, perhaps, our descriptive statistics presented early in the Results. We have left the basic description of the data in the Results section called Inquiline Communities because we feel that information describing the species composition, richness, etc. are meaningful data to present and provide basic data useful to future study of these species. We also retain data about site differences in flowering and pitcher count, but consolidate those data in Table 5, allowing us to streamline the text. We have removed the section Spatial and Temporal Variation and placed some of its former content in the section Population Size and Isolation, while removing the remaining content.

II. Some other comments

*1. The authors need to define rosettes, clumps and population sizes. Individual pitcher plants are comprised of one or more meristems – does a rosette correspond to a meristem or a plant that is composed of one or more meristems? Are clumps individual plants composed of one or more meristems or groups of plants and meristems that are too dense to separate into their component parts without damaging the plants (this is a genuine, practical concern, but if that is their concern, they need to state it in their definitions).*

We clarify our definition of clump in lines 151-156. The purpose of using these clump data were to obtain an estimate of the number of colonizable pitchers – in a metacommunity context, the number of patches – at a site. In the original manuscript, we emphasized that the number of clumps likely does not equal the number of genetically distinct individuals in the population. We stated this because we were trying to make the point that clump count is not the same as traditional population size. In reflection, we felt this point was tangential to the more relevant issue that we are using “population size” in this study to indicate the number of colonizable pitchers/patches. To resolve this, we obtained an operational definition of “clump” from the US Fish and Wildlife Service employees who conducted the 2012-3 survey and we state that we use number of clumps as a correlate total pitchers in the population.

We clarify our definition of a rosette in lines 175-178. A rosette was a single meristem from which multiple pitchers, pitcher buds, or flower buds were evident. This definition is now justified in the text. We also noticed our vague use of “plant” in a few places and revised these to “rosette”.

*They define population size by number of “the number of distinct clumps of rosettes. Although clumps may include multiple, genetically distinct plants, we assume that the number of clumps is correlated with the number of genetically distinct plants… ” (lines 140-142). So the appropriate within site-month-year independent unit is “clump,” even though census data between months or years within a clump are not independent, normalized clump data are more independent that rosettes within a clump (Lines 253-254) or leaves within a rosette.*

The reviewer raises a number of important and helpful issues here. First, we have clarified that only at site A were multiple rosettes from the same clump sampled. Therefore, we feel justified in keeping rosette as the appropriate random variable in our mixed effect models. Indeed, the three pitchers sampled from each rosette are not independent, which is why we have now averaged number of organisms and Shannon’s H within rosettes in our analyses of flowering and pitcher number.

Our experimental design is nested, with 3 pitchers nested within rosette, and rosettes nested within site. We struggled with how to test our hypotheses given this design. For the analyses of flowering and pitcher number, we have three measures of community (three pitchers) for each measure of whether a rosette flowered and how many pitchers the rosette produced. For this analysis, the appropriate nested model might be to include random effects of pitcher within rosette and rosette within site. We opted to retain site as a fixed effect and to eliminate the nested aspect of the design by averaging the three pitchers per rosette.

For the comparison of communities with plant population size and population isolation, we took a different approach. First, we only measured communities in one year, so year is not a variable in this analysis. Second, we do not agree that clump is the appropriate independent unit, nor is it possible to assign our rosettes to the clumps identified the in 2013 population surveys. Those counts were not performed directly by us, but rather by an intern of M. Alexander, and the clumps were not marked in any way. We use those counts solely for an independent measure of relative population size. Further, we made every effort to only sample one rosette per apparent clump. In the revised Methods, we explain that rosettes no closer than 0.5 meters were marked and studied, except at site A (line 180). We have reanalyzed the data with rosette as the independent unit of measure.

*One meristem puts out numerous leaves but can produce zero, one or, rarely, two flowers; similarly, one plant with multiple meristems can produce zero, one, or multiple flowers. It all comes down to what is a replicate unit within a site-month-year. Multiple leaves on the same plant are not independent and meristems/plants (rosettes?) in a clump are not independent – so maybe the unit to be entered into the error term of their analyses are number of clumps, not number rosettes within clumps, and not leaves within rosettes. Lines 244-248 & 259-265 suggest that they are using individual leaves as independent estimates for the error term in their F-tests and are therefore prone to Type I error.*

We hope that our clarification of “rosette” in the Methods, in combination with our response to the reviewer’s previous point, address this concern.

*2. Line 158: What randomization procedure was used?*

In reflection, rosettes were not randomly selected but were systematically selected to be a minimum distance from one another and to have a newly opened pitcher in June of 2015. This is now clarified in the Methods (lines 174-180).

*3. Lines 181-189, 203: This study concerns numbers and diversity of arthropods exclusively. Since rotifers and unicellular eukaryotes have been also been quantified in previous studies, the reason for their exclusion should be stated. I suspect the reason is practical and within the time-frame and knowledge of student projects – and these are very real considerations. Nonetheless, at the very least, the title of the paper should be “Arthropod” or “Arthropod symbiont” instead of “Symbiont” communities.*

We appreciate that we have only sampled a portion of the communities found in pitchers and may have an incomplete count of even the arthropods. We did not identify and count smaller organisms because of the vast quantity of samples we collected, of the limited expertise of those involved, including numerous undergraduates, and of the limited funding available (prohibiting identification via genetic sequencing). To avoid misleading the audience, we have revised the title as the reviewer suggests, to specify the focus on arthropods. We also changed ‘symbiont communities” to “inquilines” to further clarify that we focus on arthropods living with the pitcher water (and not the various moths and Hymenopterans that make us of other parts of the leaves). Therefore, the new title is “Effects of arthropod inquilines on growth and reproductive effort of the purple pitcher plant (*Sarracenia purpurea* var. *montana*)”.

*4. Line 212. Why weren’t interaction terms included? Certainly R has the facility to do so.*

R has the facility to include interaction terms. We initially did not include them because of the complexity of this data set and what we perceived as the minimal additional value that would be added by evaluating whether the effect of fluid volume differs between sites, as such an effect is irrelevant to our hypotheses. More importantly, we did not evaluate all data in a single model including both years and all three months for a couple reasons. First, not all sites were sampled in all months, making the estimate of interaction effects complicated if not impossible. Second, we have the problem of lack of independence of data. Namely, we have single measures of whether or not rosettes flowered, number of flowers, and number of pitchers produced each year. These values of the three variables are the same for all three months. To repeat these values for each month and run them in the same model would inflate our sample size and degrees of freedom.

In our new analyses, we have averaged number of inquilines and Shannon’s H across pitchers from the same rosette, as explained above. *5. Lines 206-217. Here is where the interaction terms become informative. In lines 215-216, distinguishing individual rosettes (within clumps?) proved a problem and caused them to omit the site with the smallest size (Table 1; does “size” refer to pitchers, rosettes, or clumps?). Looking “for consistency across months” does not substitute for a multiple comparisons test, especially if “months within years and sites” is not specifically entered into the model. How did they quantify “look for?”*

In Table 1, we have revised the figure to use ‘Clumps’ instead of ‘Size’ as the heading for column 2. Regarding the second point, we have now removed the monthly analysis and replaced it with a single analysis in which we average community variables across pitchers from the same rosette. Therefore, our approach to look for consistency in effects across months is no longer used.

*I am not saying that analysis of their data set is straightforward, but their approach for testing their stated hypotheses with confounding years and months is unclear and the error terms in t- and F-tests appear based on number of leaves, which are not independent observations within rosettes or clumps.

I like their rationale for pursuing this study and their stated hypotheses are clear; it is making a similarly clear path to the testing of these hypotheses that makes their results equivocal. I suggest that they define their terms, break down their hypotheses as I have done, determine what is the minimum information needed to resolve those hypotheses, justify the statistical approach (stating explicitly the constraints imposed by field conditions – which are what provide substance to their study and stating explicitly what units – leaves, rosettes, clumps, sites – were used to generate the error term), and forget all ancillary observations, data, analyses, and discussion. Their concluding statement will be dicey, as their results do not support the global conclusion “that phytotelma [sic] communities benefit host pitcher plants with increased vegetative growth and reproductive effort.” I don’t doubt that this conclusion may be true in nature, but (1) the data in Table 4 do not support the conclusion of abundance of inquilines increasing growth and may support the conclusion of inquiline diversity increasing growth in some months but not others and (2) the data in Table 6 do not support the conclusion that inquiline abundance or diversity increases reproductive effort.*

We appreciate the reviewer’s careful and thorough comments and understand that our original submission lacked in directly linking our hypotheses to our analyses and results. We hope that the combination of numbering our hypotheses in the introduction, reanalyzing our data to simplify and streamline the results, and clarifying our units of replication, we have sufficiently addressed these concerns.

The reviewer notes a misspelling in ‘phytotelma’ where, presumably, ‘phytotelmata’ is preferable. Here, we used the singular of phytotelmata much as we might say ‘pond communities’ to refer to communities of organisms within ponds. Reviewing the literature for how this word is used, it appears the singular is rarely used. In rereading our manuscript, we found that we used “inquiline community”, “symbiont community”, and “phytotelma community” interchangeably. We have revised to consistently use “inquiline community” to refer to the organisms living within pitcher fluid.

The University of North Carolina at Asheville is one of the 17 institutions of The University of North Carolina and is committed to equality of educational and employment opportunity.
