# Supplementary material for: Effects of arthropod inquilines on growth and reproductive effort among metacommunities of the purple pitcher plant (Sarracenia purpurea var. montana)
Source: PLoS One. 2020 May 8;15(5):e0232835. doi: 10.1371/journal.pone.0232835 (PMC7209241; doi:10.1371/journal.pone.0232835)
Supplement: Attachment — Submitted filename: Hale et al PLOS One response 2.docx [file pone.0232835.s003.docx]

April 17, 2020

Dr. William J. Etges
Academic Editor
PLOS ONE

Re: PONE-D-19-33605

Dear Dr. Etges,

Thank you for the opportunity to revise our manuscript entitled, “Effects of arthropod inquilines on growth and reproductive effort among metacommunities of the purple pitcher plant (*Sarracenia purpurea* var. *montana*)”. We understand that the considerable changes we made to our manuscript required careful review and re-evaluation of whether we had addressed the valuable comments you and the outside reviewer provided. The reviewer’s comments are in italics and our responses are in regular typeface. We are uploading a Word document showing all revisions as well as a document with all revisions accepted, comments removed, and citation format adjusted. Further, we have revised Figure 2 (to remove color) and have uploaded the revision.

The DOI for reviewing the data on Dryad is <https://datadryad.org/stash/share/9Z3gogbGOlzisvv1uypqbG2ooz05TXM2keJw9scMcEc>

The data will be published upon the article’s acceptance.

Sincerely,

Becca Hale

Reviewer’s Comments:

*This manuscript is greatly improved in terms of the availability of their results. I worked on making up a table:*

*A	b*

*Hypothesis	2015	2016	2015	2016*

*1	r2=	ns	ns	r2=?*

*2	ns	r2=	ns	ns*

*3	ns	?	r2=	?*

*4	ns	?	ns	?*

*If one of the 16 hypotheses were true, I then tried to enter the effect size into the table. I found filling in a significant effect or not (ns) possible but tedious for H1 and H2, but not clear for subsequent entries. What was difficult or not possible to decipher was the effect size of the predictor variables (inquiline abundance and diversity) they sought to test. My doctoral professor drilled into me “I grant you there is a significant effect, but is it substantial?” With a sample size of 380 and a 1-tailed test (their hypothesis is false if there is either no significant or a negative correlation), r2 can be as low as 4.4% and still be “significant.” The major contribution of this paper is in the results since the discussion is a concatenation of equivocation (caveat lector) – none of it wrong, because each conclusion is qualified but really summing to: The number or diversity of four arthropod inquiline species can have some, probably small, effect on host plant vegetative production or flowering in some years in some bogs at some times of the year. Were I writing a review paper on pitcher-plant ecology, this statement is how I would summarize this entire paper. Even this summary would depend on how I could fill in the table above. In short, the import of this paper remains in their data from observations in nature, replicated among bogs, among times of year, and between years, not in the discussion of their results.*

We appreciate both reviewers’ comments that the value of this work may not be in our interpretation of our results, but rather in the data we provide about these small and mostly unstudied populations. When the reviewer discussed wanting to see effect size in the initial review, I interpreted the request for effect size to indicate that we should report the difference in marginal means between treatment groups and report the partial regression coefficients, as these values indicate the magnitude of the effect, as opposed to the amount of total variance explained. We did that for hypothesis 2 (lines 350-354) in our first revision, but did not report the partial regression coefficients for the other hypotheses. This was an oversight and we have added partial regression coefficients for all analyses where we conclude support for our hypotheses.

From the reviewer’s current comments, I clearly see that R^2^ values are requested as a measure of model fit. We have added R^2^ values for best-fitting models to Tables 3 and 4. For many of our models, R^2^ values exceed 0.30, which is relatively high for ecological studies in our experience. In a few places, the R^2^s were less than 1.0. In these cases, we do not interpret support for our hypotheses. We have reported R^2^ values for our Pearson correlations, where they are clearly appropriate. Our Spearman rank correlations are non-parametric and R^2^ is not relevant.

We have been careful to be consistent in reporting effect sizes in the form of estimated marginal means (least squares means) for models whether our hypotheses were either supported or partially supported, with the caveat that where we had significant interaction terms, these values cannot easily be interpreted. We report the effect size associated with hypothesis 1a (2015) in line 311, noting that because of interaction terms, this effect is difficult to interpret. We report the effect size associated with hypothesis 1b (2016) in line 316, also noting difficult interpretation due to interactions. For the other hypothesis 1-year combinations, there were not significant effects. We report the effect size associated with hypothesis 2a (2016) in lines 386-388, indicating that rosettes that flowered had, on average, 18 more organisms than those that did not. Hypothesis 2a was not supported with 2015 flowering data. Hypothesis 2b was not supported in either year. Hypotheses 3 and 4 were evaluated using non-parametric Spearman Rank Correlations, so effect sizes cannot be calculated, as neither rank size or rank abundance/diversity is a continuous variable.

The question marks for hypotheses 3 and 4 in 2016 in the reviewer’s table occur because those hypotheses were not tested in 2016. Although we were collecting community data in 2016, those data are not included in this manuscript because they were collected using different methods in order to address a different question.

We have added additional text to the Results linking our hypotheses to our statistical results and the models presented in Tables 3 (text lines 306-317) and 4 (text lines 383-402), in order to provide the clarity that the reviewer sought in creating the table, above.

We appreciate the point that p values, and statistical significance does not mean an effect is ecologically meaningful (indeed, we switched to AIC values). We believe that the relatively small magnitude of some of our effects is clear in our figures, where shallow slopes are evident, and that including the marginal means in our text shows that we are not hiding these minimal effects.

We have appreciated the opportunity additional revisions have provided us to further refine and clarify how we present and interpret our results.
